# Systemic bacteraemia in children presenting with clinical pneumonia and the impact of non-typhoid salmonella (NTS)

**DOI:** 10.1186/1471-2334-10-319

**Published:** 2010-11-04

**Authors:** Norbert G Schwarz, Nimako Sarpong, Frank Hünger, Florian Marks, Samuel EK Acquah, Alex Agyekum, Bernard Nkrumah, Wibke Loag, Ralf M Hagen, Jennifer A Evans, Denise Dekker, Julius N Fobil, Christian G Meyer, Jürgen May, Yaw Adu-Sarkodie

**Affiliations:** 1 Bernhard Nocht Institute for Tropical Medicine, Hamburg, Germany; 2 Kumasi Centre for Collaborative Research in Tropical Medicine, Kumasi, Ghana; 3 International Vaccine Institute, Seoul, South Korea; 4 University Hospital of Wales, Cardiff, UK; 5 School of Medical Sciences, Kwame Nkrumah University of Science and Technology, Kumasi, Ghana

## Abstract

**Background:**

The diagnosis and antimicrobial treatment of pneumonia in African children in the absence of diagnostic means such as x-ray facilities or microbiological laboratories relies primarily on clinical symptoms presented by the patients. In order to assess the spectrum of bacterial pathogens, blood cultures were performed in children fulfilling the clinical criteria of pneumonia.

**Methods:**

In total, 1032 blood cultures were taken from children between 2 months and 5 years of age who were admitted to a rural hospital in Ghana between September 2007 and July 2009. Pneumonia was diagnosed clinically and according to WHO criteria classified as "non-severe pneumonia" and "severe pneumonia" ("severe pneumonia" includes the WHO categories "severe pneumonia" and "very severe pneumonia").

**Results:**

The proportion of bacteriaemia with non-typhoid salmonella (NTS) was similar in children with pneumonia (16/173, 9.2%) compared to children hospitalized for other reasons (112/859, 13%). NTS were the predominant organisms isolated from children with clinical pneumonia and significantly more frequent than *Streptococcus pneumonia*e (8/173, 4.6%). Nine percent (9/101) of children presenting with severe pneumonia and 10% (7/72) of children with non-severe pneumonia were infected with NTS. Nineteen out of 123 NTS isolates (15%) were susceptible to aminopenicillins (amoxycillin/ampicillin), 23/127 (18%) to chlorampenicol, and 23/98 (23%) to co-trimoxazole. All NTS isolates were sensitive to ceftriaxone and ciprofloxacin.

**Conclusion:**

In Sub-saharan Africa, sepsis with NTS should be considered in children with symptoms of pneumonia and aminopenicillins might often not be the adequate drugs for treatment.

## Background

Pneumonia is one of the most common diagnoses in African children presenting at hospitals and peripheral health centres and is the most important cause of mortality in children under five years of age [1,2]. Due to the lack of bacteriological laboratory facilities, antibiotic treatment of pneumonia is guided by presumptions based on clinical symptoms. According to the WHO-IMCI (World Health Organisation Integrated Management of Childhood Illness) amoxicillin is the first line drug for empirical treatment of pneumonia as it covers the commonly suspected organisms (e.g. *Streptococcus pneumoniae *and *Haemophilus influenzae*) [3]. However, this treatment scheme does not capture enteric bacteria and there is some evidence that Gram-negative bacteria such as *Salmonella spp*. occur more frequently among African children with septic pneumonia than expected. Among 166 Kenyan children with blood cultures positive for non-typhoidal salmonella (NTS), 46% fulfilled the clinical criteria of pneumonia according to WHO-IMCI. On the other hand, bacteraemia due to NTS was not associated with diarrhoea [4]. In other reports from East, West and Central Africa, the impact of NTS in systemic infections was recognized as well [5-16]. Moreover, NTS were among the most frequent organisms isolated from children with clinical pneumonia in some studies that were carried out before the vaccination against *H. influenzae *(Hib-vaccination) was introduced [14,17-19]. In the context of an improving coverage of Hib and pneumococcal immunisations, the impact of NTS as a cause of pneumonia will, most likely, increase considerably among African children [20].

In the present study, blood cultures were performed in children admitted to a rural hospital with pneumonia symptoms according to the WHO-IMCI definition in order to quantify the impact of systemic infections with NTS and to assess antibiotic drug resistance patterns.

## Methods

### Study site

The study hospital is located in the village Agogo in the Asante Akim North District of the Ashanti Region in Ghana, West Africa, and is the principal hospital of the district. Its catchment population encompasses approximately 80,000 people with roughly 25,000 citizens of Agogo itself. The HIV/AIDS prevalence in the area is around 1.9% according to the National AIDS Control Programme in 2009. Vaccination programmes against *H. influenzae *have been established in 2001.

### Study population

Between September 2007 and July 2009, 948 children between 2 months and 5 years of age who were hospitalized and from whom a blood culture was taken were included into the study. In total, 1032 blood cultures were taken during 1169 hospitalizations. The study complied with the "Ethical Principles for Medical Research Involving Human Subjects" as laid out in the Declaration of Helsinki and was approved by the Committee on Human Research Publications and Ethics (CHRPE) of the School of Medical Sciences, Kumasi, Ghana. Informed consent was sought from the parents or the guardians of the child after detailed explanation of the study and the study procedures.

### Clinical pneumonia, case classification

Pneumonia was classified following established WHO guidelines [21]. Children with cough or difficult breathing were classified as "pneumonia cases" if they had an increased respiratory rate of ≥ 50 per minute in children of 2 months up to 11 months of age and ≥ 40 per minute in children of 12 months up to 5 years of age. Children with symptoms of lower chest wall indrawing, nasal flaring, grunting (in young children) and/or subcostal retraction or additional signs of central cyanosis, severe respiratory distress, vomiting everything or inability to breastfeed/drink, or other danger signs were classified as "severe pneumonia" (including the WHO definitions for "very severe pneumonia" and "severe pneumonia").

### Blood cultures

1-3 ml of venous blood was taken from hospitalized children and directly injected into a Becton Dickinson (BD) BACTEC™ PEDS PLUS™/F bottle containing 20 ml of enriched broth with resin. The bottles were incubated at 35°C in a BD BACTEC™ 9050 automated Blood Culture system (BD Diagnostics, Sparks, Massachussets, USA) [22] for 5 days or until rated positive. Broth from positive bottles was directly examined by Gram stain and 20 μl were cultured further on sheep blood-agar, chocolate-agar and MacConkey agar (prepared in-house from Oxoid dehydrated media). Agar plates were incubated at 37°C for 18-24 h. Microscopical diagnosis after Gram staining was immediately reported to the clinical staff. Identification of *S. pneumoniae *was based on morphology of colonies and the optochin test. Oxacillin discs were used to determine sensitivity to penicillin. Diphteroids and propionibacteriae were classified as contaminants. If the patient was not known to be HIV positive, coagulase negative staphylococci, bacillus species and non-fermenters were usually also classified as contaminants. On the same day, antibiotic susceptibility testing was performed using the disc diffusion method with the susceptibility breakpoints suggested by the Clinical and Laboratory Standards Institute (CLSI). The following antibiotics were tested: amoxycillin/ampicillin, amoxyclav (amoxicillin & clavulanic acid), cefuroxime, ceftriaxone, co-trimoxazole, ciprofloxacin, gentamicin, tetracycline and chloramphenicol. The microbiological laboratory that was involved in the study is enrolled in a quarterly external quality assurance program in bacteriology from the National Institute for Communicable Diseases (NICD) of South Africa. If blood cultures that were taken from the same child presenting twice within 2 weeks yielded identical results, only the first result was included into further analyses.

### Data collection and statistical analysis

At admission of children, clinical data were recorded on standardized forms. Data collection was entirely embedded into the clinical routine. The admission chart contained a 4-paged admission sheet to be filled in by the admitting doctor. Double data entry was done by data entry clerks using a 4^th ^Dimension Database (^©^4D San Jose, California, United States). Data analysis was carried out using the STATA 10 software (College Station, Texas, United States).

For each pathogen, the number of isolates and their proportion among all blood cultures performed was calculated separately for children fulfilling the case definition for pneumonia, severe pneumonia and for non-cases.

## Results

Of the 1032 blood cultures included, 734 (71%) did not yield bacterial growth and 90 (9%) were positive with non-pathogenic microorganisms (contaminants). Pathogens were isolated from the remaining 208 (20%) blood cultures. Antiobiotic pre-medication was reported from 7% of children and was independent of the positivity of blood cultures. The frequency of children without pneumonia (12.2%), pneumonia cases (8.9%) and severe pneumonia cases (9.8%), was similar in children with available and with missing blood cultures (p = 0.5, χ^2 ^= 1.2).

In total, 173 children fulfilled the case definition of pneumonia of any severity. Blood cultures were positive in 18.1% (13/72) of children with non-severe pneumonia and in 26.7% (27/101) of children with severe pneumonia. The frequency of bacteraemia was similar high in the 859 children without pneumonia (168/859, 19.6%). Ten children with severe pneumonia died: 2 children with a systemic NTS infection (1 with aminopenicillin resistant strains), 3 with *S. pneumoniae *bacteraemia, 1 with *S. *Typhi, and 4 with negative blood cultures. Fatalities were not observed among the patients with non-severe pneumonia.

Among all children with and without bacteraemia, 24% and 34% were positive for malaria parasites, respectively. Children with NTS bacteraemia had more often a co-infection with malaria parasites (24%) compared to those with *S. pneumoniae *bacteraemia (18%). Malaria co-infection was found in 35% of non-cases, 28% of pneumonia cases and 22% of severe pneumonia cases.

In patients without pneumonia, NTS represented 66.7% (112/168), *S. aureus *10.1% (17/168), *S. pneumoniae *7.1% (12/168) and *S. *Typhi 6.5% (11/168) of the pathogen isolates. In non-severe pneumonia cases NTS represented 53.8% (7/13), *S. aureus *7.7% (1/13), *S. pneumoniae *15.4% (2/13) and *S. *Typhi 7.7% (1/13) of the pathogen isolates. In severe pneumonia cases NTS represented 33.3% (9/27), *S. aureus *14.8% (4/27), *S. pneumoniae *22.2% (6/27) and *S. *Typhi 11.1% (3/27) of the pathogen isolates. When looking at all pneumonia cases of any severity, 16 of 40 (40%) of all pathogen isolates were NTS (Table 1).

**Table 1:** Blood culture results in children between 2 months and 5 years of age

	All pneumonia n = 173		Non-severe pneumonia* n = 72		Severe pneumonia n = 101		No pneumonia n = 859		Total n = 1032	
	n	%		%	n	%	n	%	n	%
NTS	16	9.3	7	9.7	9	8.9	112	13.0	128	12.4
*S*. Typhi	4	2.3	1	1.4	3	3.0	11	1.3	15	1.5
*Shigella *spp.	0	-	0	-	0	-	3	0.4	3	0.3
*Enterococcus *spp.	0	-	0	-	0	-	1	0.1	1	0.1
*S. pneumoniae*	8	4.6	2	2.8	6	5.9	12	1.4	20	1.9
*H. influenzae*	0	-	0	-	0	-	1	0.1	1	0.1
*Streptococcus *spp.	1	0.6	1	1.4	0	-	3	0.4	4	0.4
*S. aureus*	5	2.9	1	1.4	4	4.0	17	2.0	22	2.1
*Klebsiella *spp.	2	1.2	1	1.4	1	1.0	2	0.2	4	0.4
*Pseudomonas *spp.	1	0.6	0	-	1	1.0	0	-	1	0.1
*Acinetobacter *spp.	1	0.6	0	-	1	1.0	2	0.2	3	0.3
*M. morganii*	1	0.6	0	-	1	1.0	0	-	1	0.1
*E. coli*	1	0.6	0	-	1	1.0	4	0.5	4	0.5
Contaminant	11	6.4	5	6.9	6	5.9	79	9.2	90	8.7
Negative	122	70.5	54	75.0	68	67.3	612	71.3	734	71.1

* Pneumonia cases according to WHO-IMCI definition. Agogo Presbyterian Hospital 09/2007-07/2009

# Severe pneumonia included the WHO definitions for "very severe pneumonia" and "severe pneumonia"

### Sensitivity to antibiotics

All NTS and *S. pneumoniae *isolates from children aged 2 months to 5 years were included for sensitivity testing against 9 antibiotics irrespective of their clinical diagnosis (Table 2). 15% of all NTS isolates were sensitive to aminopenicillins (amoxycillin/ampicillin), 18% to chloramphenicol, 23% to co-trimoxazole, and 71% to gentamicin. All isolates of NTS were susceptible to ceftriaxone and ciprofloxacin. The frequency of multidrug resistance (MDR, resistance against amoxycillin, chloramphenicol, and co-trimoxazole) was 75.5% (74/98).

**Table 2:** Proportion susceptible to antibiotics among NTS *and S. pneumoniae *isolates

	NTS* (N = 128)		S. pneumoniae* (N = 20)	
	**% susceptible**	**n tested**	**% susceptible**	**n tested**

Amoxycillin/Ampicillin	15.5%	123	80.0%	20
Amoxyclav	25.7%	113	88.9%	18
Cefuroxime	46.5%	127	100%	6
Ceftriaxone	100%	108	100%	14
Co-trimoxazole	23.5%	98	0%	17
Ciprofloxacin	100%	127	52.6%	19
Gentamicin	70.9%	127	22.2%	18
Tetracyclin	89.0%	91	25.0%	16
Chloramphenicol	18.1%	127	88.9%	18

*Blood cultures from children aged 2 months to 5 years of age. Agogo Presbyterian Hospital 09/2007-07/2009

## Discussion

In the present study, non-typhoid salmonella (NTS) were found in 10% of the blood cultures and were the predominant cause of bacteraemia in hospitalized children. This prevalence is, most likely, underestimated due to the small blood volumes that were available in the paediatric patients and the frequent self-medication with antibiotics prior to blood sampling. It has been reported that the sensitivity of blood cultures fell by almost one third if a blood volume of 1 ml was inoculated to blood culture bottles compared to a volume of 3 ml as recommended [23].

The frequency of NTS was similar among children with pneumonia and those without pneumonia and it cannot be determined whether bacteraemia due to NTS is the exclusive cause of pneumonia or predisposes to secondary infections with other microorganisms. However, from a practical point of view this may be irrelevant: if NTS occur frequently among children with severe respiratory symptoms, empirical treatment of pneumonia must include coverage of NTS.

In Africa, aminopenicillins or co-trimoxazole are currently recommended for empirical treatment of community-acquired pneumonia in children, assuming that the most frequent causal infectious organisms are *S. pneumoniae *and *H. influenzae*. The IMCI-WHO guidelines recommend treating non-severe pneumonia with cotrimoxazole or amoxicillin given orally, severe pneumonia with oral amoxicillin or injectable penicillin and very severe pneumonia with injectable ampicillin plus injectable gentamicin [3].

Empirical treatment of childhood pneumonia with the aforementioned first line drugs may readily fail due to the high frequency of drug resistance of NTS [20]. In the present study, only 15.5%, 23.5% and 70.9% of NTS isolates were sensitive to aminopenicillins, co-trimoxazole and gentamicin, respectively. Multi-drug resistance against the three standard drugs amoxicillin, chloramphenicol and co-trimoxazole was 75.5%. High levels of MDR have also been reported from the Democratic Republic of Congo [24], Malawi [8,25], The Gambia [26,27] and Kenya [28]. A study from Tanzania has demonstrated strong association of antimicrobial resistance with fatal courses of children with bloodstream infections [29]. Fifteen years ago, no resistance to chloramphenicol and 57% resistance to ampicillin was reported in NTS collected from children in Accra, Ghana [16]. A more recent study from Accra found MDR in 87% of *Salmonella *serogroup B isolates [30]. In Northern Ghana, among 6 isolates of *Salmonella spp*. from children with diarrhoea, only one was resistant to ampicillin and chloramphenicol [31]. This demonstrates that marked local and temporal differences of antibiotic resistance patterns of NTS exist [32].

Ceftriaxone or ciprofloxacin may be appropriate alternatives to treat bacterial infections causing pneumonia in areas with prevalences of NTS. Notably, 50% of the pneumococcal isolates were resistant to ciprofloxacin in the present study compared to only 4% of all pneumococcal and NTS isolates in 1997 [33]. However, the use of ceftriaxone in low-resource countries has considerable disadvantages which have to be considered: (i) the drug has to be adminstered intravenously or intramuscularly; (ii) well trained staff is necessary; (iii) uncritical use may promote the development of resistance; (iv) ceftriaxone is approximately 3 to 10 times more expensive than amoxicillin.

## Conclusion

Aminopenicillins may often not be adequate for the treatment of children presenting with pneumonia symptoms in Sub-saharan Africa and NTS in children with pneumonia should strongly be considered as possible infectious agent. The development of alternative drugs and, best, vaccines against NTS as well as appropriate surveillance of this neglected infectious disease is necessary.

## Competing interests

The authors declare that they have no competing interests.

## Authors' contributions

Authors contribution: JM, CGM, JAE, and YAS planned and initiated the study. NS organised the day-to-day work as study coordinator. FH planned and established the microbiological laboratory facilities at the Agogo Presbyterian Hospital. SEKA, AA and BN carried out the microbiological cultures and antimicrobial resistance testing. DD substantially contributed to microbiological work-up and the preparation of the manuscript. RMH, JF and FM contributed to the analysis and the writing of the manuscript. WL created Case Record Forms and was responsible for data management and data preparation for analyses. NGS designed the analysis protocol and analysed the data. JM, NGS, and CGM wrote the manuscript. All authors have read and approved to the final version of this manuscript. Parts of this research have been financially supported by a Swiss Foundation.

## Pre-publication history

The pre-publication history for this paper can be accessed here:

http://www.biomedcentral.com/1471-2334/10/319/prepub
